# Maximising recombination across macadamia populations to generate linkage maps for genome anchoring

**DOI:** 10.1038/s41598-020-61708-6

**Published:** 2020-03-19

**Authors:** Kirsty S. Langdon, Graham J. King, Abdul Baten, Ramil Mauleon, Peter C. Bundock, Bruce L. Topp, Catherine J. Nock

**Affiliations:** 1 Southern Cross Plant Science, Southern Cross University, Lismore, NSW Australia; 2 AgResearch NZ, Grasslands Research Centre, Palmerston North, New Zealand; 3 Queensland Alliance for Agriculture and Food Innovation, University of Queensland, Brisbane, Qld Australia

**Keywords:** Plant sciences, Plant genetics

## Abstract

The Proteaceae genus *Macadamia* has a recent history of domestication as a commercial nut crop. We aimed to establish the first sequence-based haploid-correlated reference genetic linkage maps for this primarily outcrossing perennial tree crop, with marker density suitable for genome anchoring. Four first generation populations were used to maximise the segregation patterns available within full-sib, biparental and self-pollinated progeny. This allowed us to combine segregation data from overlapping subsets of >4,000 informative sequence-tagged markers to increase the effective coverage of the karyotype represented by the recombinant crossover events detected. All maps had 14 linkage groups, corresponding to the *Macadamia* haploid chromosome number, and enabled the anchoring and orientation of sequence scaffolds to construct a pseudo-chromosomal genome assembly for macadamia. Comparison of individual maps indicated a high level of congruence, with minor discrepancies satisfactorily resolved within the integrated maps. The combined set of maps significantly improved marker density and the proportion (70%) of the genome sequence assembly anchored. Overall, increasing our understanding of the genetic landscape and genome for this nut crop represents a substantial advance in macadamia genetics and genomics. The set of maps, large number of sequence-based markers and the reconstructed genome provide a toolkit to underpin future breeding that should help to extend the macadamia industry as well as provide resources for the long term conservation of natural populations in eastern Australia of this unique genus.

## Introduction

The Gondwanan plant family Proteaceae comprises 83 genera and over 1,600 species^1^. *Macadamia* was the first genus in the family to be domesticated although others are also cultivated for edible kernel (*Gevuina*) or for ornamental use (e.g. *Protea*, *Telopea*, *Banksia*). The long-lived Australian subtropical rainforest trees *M. integrifolia* (Maiden & Betche) and *M. tetraphylla* (L.A.S. Johnson) and their hybrids underpin the macadamia ‘nut’ industry. Although commercially important, there is limited understanding of genome structure and the genetic basis of adaptive traits^2^. Macadamia is diploid, with a reported haploid chromosome number of 14^3,4^. Although mass flowers are produced on racemes, less than 5% of these set fruit^5^, and partial self-incompatibility has been observed^6,7^. World macadamia production has expanded faster than any other tree nut crop over the past ten years and currently accounts for ~3% of the global nut trade^8^. There is growing need to develop new cultivars to meet yield and quality standards, as well as increased cultivation for a wider range of cultivation conditions^9^. The ability of breeders to improve yield, pest and disease resistance, and tolerance to a variety of environmental conditions is greatly enhanced by aligning trait loci and underlying genetic variation with the genome^10^.

Genetic linkage maps are a powerful resource that can improve breeding efficiency by identifying the relative chromosomal location of genes underlying key phenotypic traits, and assisting in the development of selective markers, which may be of particular value in perennial crops with long generation times. More specifically, dense linkage maps remain valuable for anchoring and orienting whole-genome sequence scaffolds, mapping of quantitiative trait loci (QTL), and informing comparative genomic and evolutionary studies^11–14^. Given that linkage maps represent the distribution of chiasmatic crossovers (COs) resulting from parental meiotic recombination, they provide valuable insights into the contribution that each set of parental alleles may make to subsequent progeny. They may also be used for map-based cloning, to estimate the rate of introgression of specific alleles, and to determine the likelihood of linkage drag between loci^15^.

It is widely accepted that the marker order and the length of genetic linkage maps can vary within species, and the notion that a single linkage map represents an accurate distribution of variation in recombination for a species is flawed^16^. Inconsistencies in marker order between maps are inevitable as each map is based on the segregation of alleles resulting from independent parental meioses that arise from the particular cross used to generate the map.

Many early linkage maps for a range of species were constructed using a limited number of molecular markers, and were often not of sufficient density or resolution to achieve fine mapping of QTLs, MAS or genome anchoring^17^. The introduction of next generation DNA sequencing (NGS) and genotyping by sequencing (GBS) marker generation has led to the production of many revised linkage maps with increased marker density and resolution that has improved the downstream applications of linkage maps.

The first macadamia genetic linkage maps were constructed using 328 arbitrary amplified RAF and RAPD markers and a single tagged STMS marker from 56 F_1_ progeny of HAES ‘246’ x HVP ‘A16’^18^. However, due to low marker density and a lack of sequence-based markers they were unable to be used for QTL detection or genome anchoring. This highlights the need for robust, dense linkage maps developed using sequence-based markers to provide an accurate reflection of parental meioses, comprehensive coverage of each chromosome, and selective markers of value for future breeding programs.

A draft genome assembly of *M. integrifolia* cultivar HAES ‘741’ has been generated, consisting of 193,493 scaffolds with a combined length of 518 megabases (Mb)^2^. Following subsequent improvements, the most recent ‘741’ assembly comprises 4,098 scaffolds with a combined length of 744 Mb^19^. However, there is no information on the relative location or orientation of these scaffolds with respect to the individual chromosomes karyotype.

There is evidence that SNP-based genetic linkage maps from multiple populations can, in combination, maximise genome anchoring and orientation^20,21^. We compared the ability of multiple parental maps and integrated cultivar-specific maps to increase marker density and accuracy of marker order when compared with individual linkage maps. We also explored parental contributions to observed variation in recombination rate and the relative reliability and congruence of individual and integrated maps. Overall, this approach allowed us to develop a series of genetic linkage maps which captured the range of recombination from four populations and provided a suitable density of sequence based markers to anchor genome assembly sequences. To our knowledge, these platform genomic resources are the first sequence-based haploid-correlated genetic linkage and physical maps for macadamia, and will contribute significantly to future breeding programs and macadamia research.

## Material and Methods

### Plant materials

**Figure 1:**
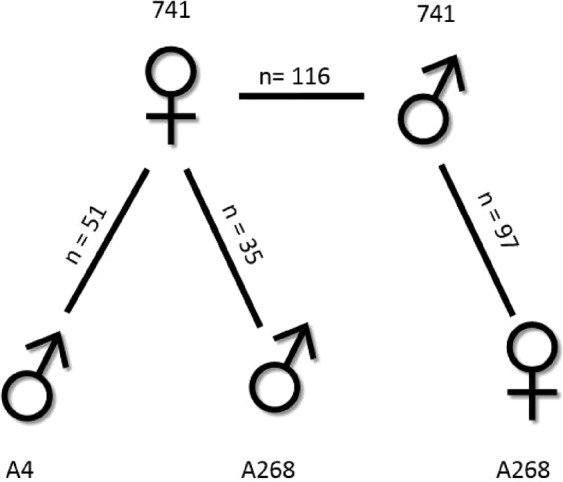
*Macadamia* populations used to create genetic linkage maps in this study. Figure shows the maternal and paternal cultivars and number of progeny for each cross.

Four mapping populations were established using self and biparental crosses of macadamia cultivars (Fig. 1) with a common *M. integrifolia* parental cultivar (‘741’). This cultivar had previously been used as the reference genotype for genome sequencing and assembly^2^ and originated from the Hawaiian Agricultural Experiment Station (HAES). Populations comprised unselected progeny from self-pollinated ‘741’, as well as biparental crosses of ‘741’ and the *M. integrifolia* - *M. tetraphylla* hybrid cultivars ‘A268’ and ‘A4’ from Hidden Valley Plantations (HVP), Australia (Fig. 1). These crosses were selected to reflect parental differences in a range of economically important traits^22,23^. The populations were maintained at the Queensland Department of Agriculture Maroochy, Nambour, and Bundaberg Research Facilities, and at Southern Cross University, Lismore, Australia.

Cultivar ‘741’ was selected from the Hawaiian seedling orchard Deschwanden and its parentage is unknown^24^. Previous genetic evidence indicates that ‘741’ is pure *M. integrifolia*^25,26^. Cultivar ‘A268’ is an F1 hybrid of *M. integrifolia* and *M. tetraphylla* and ‘A4’ is also a hybrid with approximately 25% *M. tetraphylla* content. The parents of ‘A4’ are the F1 *M. integrifolia – M. tetraphylla* hybrid ‘Renown’ and *M. integrifolia* ‘Own Choice’^22,27^.

Approximately one gram of young leaf material was collected from at least 2 replicates of each parental cultivar and from each individual within the four mapping populations. The leaf material was dried with 10 times wet weight of self-indicating silica gel and sealed in individual resealable plastic bags. Samples were stored in sealed plastic containers at room temperature with hydrated silica gel replaced as required.

### DNA extraction

Total genomic DNA was extracted using a DNeasy Plant Mini Kit or DNeasy 96 Plant Kit (Qiagen Inc. Valencia, USA) from approximately 20 mg of dry leaf material following methods previously reported^7^.

Mature, sclerophyllous leaf material was processed in individual tubes as the required concentration and quality of DNA was difficult to obtain using the DNeasy 96 microtiter plate method. In some cases, individual extracts or pooled multiple extractions were concentrated using an Eppendorf Concentrator 5301 (Eppendorf AG, Hamburg) in order to obtain minimum concentration, 50 ng/µl, required for downstream analysis. For a small number of samples multiple lysates were loaded onto columns to increase concentration. DNA was quantified using a NanoDrop 2000/2000c spectrophotometer (Thermo Fisher Scientific Inc) and a Qubit dsDNA BR assay (Life Technologies, Carlsbad, USA).

### Generation of marker data

Marker genotyping was conducted by Diversity Array Technologies Ltd. using DArTseq methodology^28^. DArTseq is is a rapid, cost-effective platform for genetic marker discovery and genotyping that targets non-repetitive regions and has been used extensively for genetic linkage mapping of plant crops^29^. Methods followed those previously optimized and described for macadamia using *PstI* + *HhaI* genome complexity reduction to generate co-dominant SNP and dominant presence-absence variant (PAV) sequence markers of 64 base pair (bp) in length^30^.

### Marker filtering

Exclusion of markers from the data set was based on the following parameters. Markers were excluded if there was: inconsistency between biological or technical replicates of parental genotypes, or greater than 10% missing values in progeny, or ambiguous or impossible segregation (had allelic combinations inconsistent with Mendelian segregation from the respective parental gametes). Quality control parameters provided by DArT, as outlined by Barilli, *et al*.^29^ were applied, and markers with reproducibility >90%, call rate >90% and average polymorphic information content (PIC) > 0.25 were retained for genetic linkage mapping.

### Linkage map construction

The program JoinMap 5.0^31,32^ was used to construct all linkage maps. A ‘741’ self map was constructed using heterozygous SNP markers coded for a cross pollination (CP) population. Phase information generated in JoinMap for each marker was then used to recode markers for an F2 population. An F2 map was generated using the multipoint likelihood mapping algorithm, the Kosambi mapping function with default settings, recombination frequency calculated with a maximum of 200 iterations, and a recombination tolerance of 1.0 e^−0.8^. Markers that showed significantly distorted segregation (P < 0.01) were removed if adjacent markers segregating for the same parent did not show distortion.

Maternal and paternal maps were constructed using either filtered segregating SNP and PAV markers for the biparental crosses (‘741’ x ‘A4’), (‘741’ x ‘A268’) and (‘A268’ x ‘741’). These maps were generated for each parent individually in JoinMap using the parameters already described.

Map integration for the four ‘741’ maps and two ‘A268’ maps was performed using the regression mapping algorithm with default settings in JoinMap.

In order to determine marker order consistency across the maps, Spearman’s Rank correlation coefficients were calculated. Each of the parental ‘741’ maps was compared to the ‘741’ self map, while both the ‘A268’ maps were compared to the ‘A4’ map to test for map congruence.

Marker distortion was estimated by Chi–square goodness-of-fit test with a threshold p-value of 0.01 in JoinMap. The number of SNP markers showing segregation distortion was counted for each of the parental maps and the ‘741’ self map. MapChart v2.3^33^ was used to illustrate regions of segregation distortion (SD) on the ‘741’ self-map.

### Crossover frequency

The number and distribution of apparent crossovers (COs) was assessed by visual interrogation of the colour coded (graphical) JoinMap genotyping data output. Apparent COs, based on the number of observed exchanges for each genotype, were recorded for all progeny in each linkage group across the six parental maps. Individuals with no apparent COs per linkage group and proportion of distal COs (within the end 10% of linkage group length) were also recorded.

### Scaffold anchoring

ALLMAPS is a method that configures sequence scaffolds to maximise the collinearity of markers located on physical and genetic linkage maps^34^. Multiple linkage maps can be included in a single run and scaffolds are ordered and orientated to generate sequences that are concordant with the input linkage maps.

To create the chromosome-scale physical genome assembly of macadamia, genetic linkage map information was used to order and orient the 4,098 scaffolds from the *M. integrifolia* ‘741’ v2 genome assembly [European Nucleotide Archive (EMBL-ENA) repository, assembly accession: ERS2953073] using ALLMAPS software^34^. The software required two datasets as input: (1) the genetic position in centimorgans (cM) of markers on all nine maps, and (2) the physical location of markers used in the genetic map on the v2 genome assembly, obtained via marker sequence alignment to the scaffolds. These datasets were tabulated and saved as a comma-separated value text files for use by ALLMAPS. The ALLMAPS output generated for each map provided ordered and anchored scaffolds for each linkage group and identified disagreements in marker order between linkage maps.

A consensus physical map was produced in ALLMAPS, using the *merge* function from the linkage information of the ‘741’ self map and the six parental maps. This integrated the scaffold order based on marker order from the input maps, and increased the number of markers available to order and anchor genome scaffolds. Four different weighting schemes were tested. The optimal weighting regime was selected based on map confidence, congruence and number of anchored and orientated scaffolds. Chromosomal scale sequence was constructed in ALLMAPS with adjacent scaffolds joined with gaps of 100 Ns. Maps were merged with the following weights applied: weight 3 for the ‘741’ self-map, weight 2 for parental ‘741’ maps and weight 1 for the ‘A4’ and ‘A268’ hybrid cultivar parental maps. Linkage groups were subsequently ordered into pseudo-chromosomes based on congruence between genetic and physical maps, relative size in megabases and consistency of marker distribution across maps.

In order to determine the relationship between genetic and physical location of the markers from the genetic linkage maps, marker sequences were aligned this time against the pseudo-chromosomes of the consensus map generated by ALLMAPS, with marker position determined at alignment thresholds of ≥80% query coverage, >90% sequence identity, bit score ≥40 and e-value <1e^−6^. The genetic position of markers on both the integrated ‘741’ map and the integrated ‘A268’ map were then plotted against the physical location in base pairs (bp) of the markers along the same pseudo-chromosome.

## Results

### Mapping populations

Progeny were assigned to mapping populations based on paternity analysis using DArT SNP markers and methods previously described^7^. The proportion of progeny not assigned to the expected cross ranged from 5.6% to 43.3% (Table S1). Self-fertilisation was detected in both the ‘741’ x ‘A268’ (3.3%) and the ‘741’ x ‘A4’ (5.6%) populations but not in the ‘A268’ x ‘741’ population. A subset of 299 individuals from four distinct populations was selected for mapping (Fig. 1).

### Marker information

Unfiltered data from 6,071 SNP markers and 10,071 PAV markers were obtained for the four populations. Markers were first filtered to remove those discordant in genotype between the parental replicates. Within the three biparental populations, 47% of informative SNP markers suggested impossible segregation (had allelic combinations inconsistent with Mendelian segregation from the respective parental gametes) and were excluded from further analysis. In addition, between 3–25% of markers had missing data and a further 7.5–24% were removed based on PIC, reproducibility or call rate. After quality filtering to remove low quality markers 901 (‘741’ self), 3,805 (‘741’x’A4’), 3,533 (‘741’x ‘A268’), and 2,650 (‘A268’ x ‘741’) informative markers were identified for the respective populations (Table S2).

### Segregation types

The ‘741’ self-map was constructed from the largest population (n = 116) using heterozygous SNP markers. The biparental populations allowed independent investigation of male and female meioses. Similar proportions (38–40%) of pre-filtered markers were polymorphic in each of the populations, with similar SNP heterozygosity within the parents of 0.18 (‘741’ and ‘A268’) and 0.16 (‘A4’). After quality filtering, between 2–4% of available markers in the biparental populations segregated with a co-dominant F2 ratio of 1:2:1, 16–18% segregated with a backcross ratio of 1:1 and 80% segregated with a dominant 3:1 ratio. Overall, 20% of the filtered markers available for mapping were SNP and 80% were PAV (Table S2). Approximately twice the number of markers were in coupling compared to repulsion phase for ‘741’ and ‘A4’, while 25% of markers were in coupling for ‘A268’ (Table S3).

### Map construction

#### Self and parental linkage maps

Access to segregation data from different types of populations provided an opportunity to develop a range of maps, capturing recombination behaviour in different contexts. In total, nine genetic linkage maps were constructed, including one self, three maternal, three paternal, and integrated maps for each of the cultivars ‘741 and ‘A268’ (Table 1). Each map comprised 14 linkage groups, consistent with the haploid chromosome number of *Macadamia*^3^. The ‘741’ self-map was constructed exclusively with codominant SNP markers, whilst the parental maps incorporated both SNP and PAV markers.

**Table 1:** Summary statistics for *Macadamia* genetic linkage, integrated and ALLMAPs consensus maps and genome anchoring.

Map	n	Analysis	Total map length	Linkage group length		No. Markers	Unique Map Positions	Largest Gap	Marker Interval	Genome assembly anchored	Scaffolds anchored	N50 Scaffolds anchored	Scaffolds> than 1 marker
			cM	Minimum cM	Maximum cM			cM	Marker positions/cM	%			
Map 1 Self SNP (741×741)	116	JoinMap (F2)	846.7	47.8	72.0	884	555	12.5	0.66	33.3	550	246	176
Map 2 **741**♀ (741xA4)	51	JoinMap (CP)	1227.0	52.7	159.6	1210	565	16.1	0.46	39.9	687	296	228
Map 3 **741**♀ (741xA268)	35	JoinMap (CP)	985.5	23.9	127.7	877	365	21.0	0.37	32.8	543	240	158
Map 4 **741**♂ (A268x741)	97	JoinMap (CP)	1906.4	62.7	208.6	694	568	31.2	0.30	29.0	447	213	121
Map 5 **A4**♂ (741xA4)	51	JoinMap (CP)	1901.7	75.1	233.7	1929	852	31.8	0.45	50.4	894	374	381
Map 6 **A268** ♂ (741xA268)	35	JoinMap (CP)	1261.7	17.9	130.5	1797	525	24.3	0.42	46.9	820	355	336
Map 7 **A268** ♀ (A268x741)	97	JoinMap (CP)	3662.1	188.3	520.4	1701	1210	39.1	0.33	46.4	803	351	318
Map 8 **741** Integrated		JoinMap (integrated)	835.8	50.6	72.6	1445	1277	13.9	1.53	42.8	754	314	279
Map 9 **A268** Integrated		JoinMap (integrated)	1029.8	65.1	92.1	2084	1991	20.3	1.93	51.7	920	389	389
ALLMAPs Consensus		ALLMAPs (Consensus)				4184				69.7	1465	474	856

Number of progeny used in map construction (n), population parameters used in JoinMap F2 or CP, genetic distance in centimorgans (cM), number of markers mapped to a unique position.

Map lengths ranged over four-fold, from 847 cM to 3,662 cM, with between 365 and 1,210 markers mapping to distinct loci (Table 1). The paternal map was the longest of the ‘741’ maps (1,906 cM) with most markers (568) mapping to a unique position. Mean marker intervals ranged between 0.30 and 0.5 markers per cM in the parental maps, but was highest (0.66) in the ‘741’ self map. Some linkage groups (LG11 and 12 on Map 3, LG13 on Map 2 and LG01 on Map 4) included fewer markers. In comparison, the hybrid parental maps were longer, ranging from 1,262 cM (paternal ‘A268’) to 3,662 cM (maternal ‘A268’). The ‘A4’ map included the most markers (1,929) although only 852 of these mapped to unique locations in comparison to 1,210 unique locations for markers on the ‘A268’ maternal map. Marker density of the hybrid parental maps ranged from 0.33 to 0.45 markers per cM (Table 1).

### Map integration increases marker density

**Figure 2:**
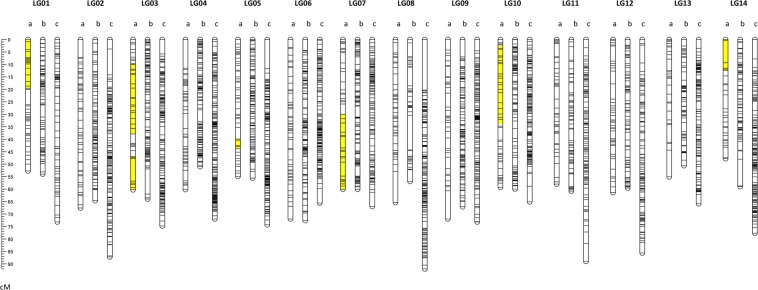
Marker distribution in genetic linkage maps. (**a**) Map 1 (741 ×741), (**b**) Map 8 (integrated ‘741’) and (**c**) Map 9 (integrated ‘A268’). Yellow sections on Map 1 indicate the areas of segregation distortion. Scale in centimorgans (cM). Linkage group (LG).

The integration of markers from multiple parental maps can increase both the number of markers available and marker density. Parental maps were first investigated and homology of linkage groups between maps was determined based on common markers, allowing for the data from the relevant linkage groups in the separate populations to be integrated (Table 1). Integrated maps for cultivars ‘741’ and ‘A268’ incorporate segregation information from constituent maps and resulted in higher marker density compared to the component maps. The integrated maps included the largest number of markers and overall marker densities (Table 1, Fig. 2).

Although the length of the integrated ‘741’ map (Map 8) was similar to each of the ‘741’ maternal maps, it included a total of 1,445 markers, of which 1,277 (88%) represented unique map positions. This resulted in a higher marker density (1.5 markers per cM) than the parental ‘741’ maps (0.30, - 0.46) and the ’741’ self map (0.66). Integrating both parental ‘A268’ maps reduced the map length (1,030 cM) by 28%, and increased the marker density to 1.93 markers per cM in comparison to 0.33 and 0.42 markers/cM for the parental ‘A268’ maps (Table 1).

### Map congruence supported marker order

Congruence of marker locus order between maps is an indication of map reliability and accuracy of order. Congruence was determined using Spearman’s Rank correlation (ρ) (Table S4) and was high between the ‘741’ self (Map 1) and ‘741’ maternal map (Map 2, average across linkage groups ρ = 0.93, n = 16, p < 0.01). The lowest level of congruences (ρ = 0.77, n = 9, p < 0.05) was observed between Map 1 and the paternal ‘741’ map (Map 4). This appeared to be due predominantly to three linkage groups carrying a low number of common and tightly linked markers (LG10, 0.44, LG11, 0.2 and LG12, 0.0). Linkage group 05 of the ‘741’ maternal map (Map 3) also had low congruence with Map 1 (ρ = 0.22, n = 16, p > 0.05). As expected, the ‘741’ integrated map (Map 8) had complete congruence (ρ = 1.0, n = 59, p < 0.01) with Map 1.

For the hybrid maps congruence was highest (ρ = 0.82, n = 37, p < 0.01) between the ‘A4’ paternal map (Map 5) and the ‘A268’ paternal map (Map 6). However, for LG12 a correlation could not be established due to a lack of common markers. Average congruence was higher (ρ = 0.8, n = 99, p < 0.01) between Map 7 and Map 6 than between Map 5 and Map 7 (ρ = 0.75, n = 30, p < 0.01). Where congruence was low it reflected the smaller number of tightly linked markers on one of the maps (Fig. 1, Table S4).

### Segregation distortion

The ‘741’ self map (Map 1) was particularly valuable for detecting variation in SD, as it was constructed exclusively using co-dominant SNP markers. Regions of SD were identified using the Chi-squared test to detect departures from expected Mendelian segregation (Fig. 2). In total, 143 (16%) markers were located within seven distinct SD regions on six linkage groups, including two on LG03. The evidence for SD regions was supported at densities of at least one marker per cM, from seven markers spanning 4 cM (LG05) to 34 markers spanning 31 cM (LG10).

Based on evidence from multiple maps, it appeared that there was considerable variation in the ability of SNP markers to detect SD in different maps. The proportion of such markers varied from 1–1.5% in the ‘741’ parental maps, and increased in the ‘A268’ male (3.3%), ‘A4’ male (4.3%) and ‘A268’ female (8.8%) maps.

### Crossover frequency

**Figure 3:**
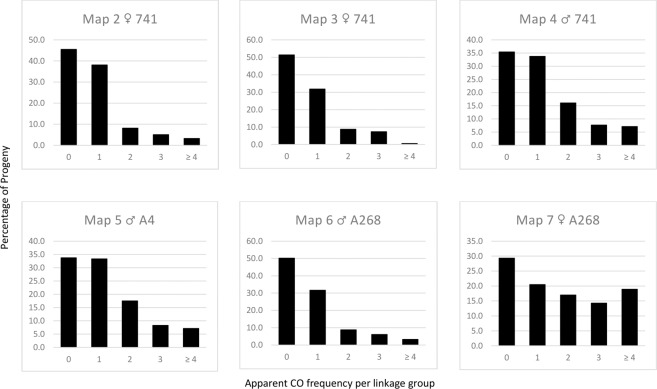
Distribution of apparent chiasmata for parental maps (Maps 2–7). The X axis is average chiasmata count per linkage group for each map. The Y axis is the percentage of progeny.

Apparent crossover (CO) frequency was calculated by counting the predicted genetic COs for each linkage group in each parental map (Fig. 3, Table S5). Across the parental maps, CO was not detected within one or more linkage groups for 29–51% of progeny. Within the meioses surveyed single CO were most common, detected in 21–38% of progeny.

In each population, counts of one or zero COs were recorded amongst the 14 linkage groups for between 50% and 84% of progeny (Table S5). Crossover counts of ≥2 per linkage group were recorded for 16 to 33% of progeny in maps generated from the biparental cross ‘741’ x ‘A4’, although none were detected on LG13 of the maternal ‘741’ map (Map 2). Both the maternal and the paternal maps from the biparental cross ‘741’ x ‘A268’ had a smaller proportion of progeny with CO counts ≥2 (17 and 18% respectively). It is worth noting there were no CO counts >1 in LG11 of Map 3 and LG01 and LG12 of Map 6. However, this may be due to the relatively small size of the ‘741’ x ‘A268’ population (n = 35).

The highest apparent CO frequency detected was in the ‘A268’ x ‘741’ population. Crossover counts ≥2 per linkage group were recorded for 31% of progeny contributing information to the ‘741’ male map (Map 4) and 50% for the ‘A268’ female map (Map 7). Crossover counts >4 were recorded for over 20% of progeny for five linkage groups in Map 7 (Fig. 3, Table S5). In addition, preliminary counts of apparent distal COs indicate that a greater proportion may be occurring in these regions for maps generated from this cross (18–20%) compared to maps generated from the other crosses (9–16%).

### Anchoring of genome scaffolds

#### Marker sequence alignment

**Figure 4:**
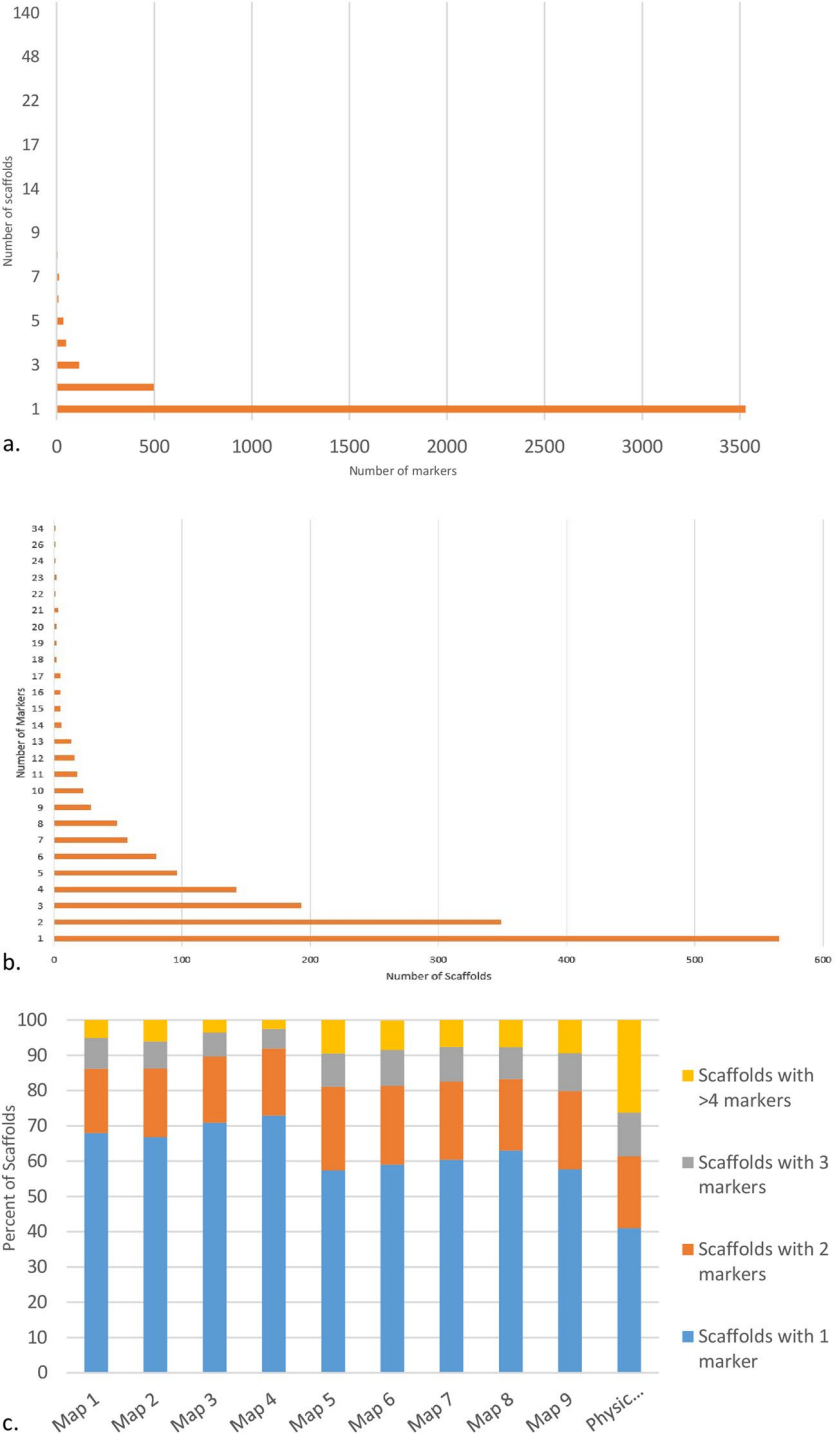
Distribution of markers mapped to scaffolds. (**a**) scaffolds per markers, ranging from one unique location to 140 different scaffolds. (**b**) markers per scaffold, ranging from one to 34 markers. (**c**) percentage of scaffolds with one or more markers for each genetic linkage map and the ALLMAPs consensus map.

Based on BLASTn analysis, a total of 4,266 marker sequences mapped to 1,667 different scaffolds, with most mapping to a single unique location within a unique genome scaffold (83%, 3,529 markers). A further 12% (512 markers) mapped to two, 3% (128 markers) to three scaffolds and 2% mapped to more than three scaffolds. Two anomalous markers, mapped to 140 and 92 scaffolds respectively (Fig. 4a), suggesting that these markers may be associated with a transposon or other repeat sequence. The distribution of markers across scaffolds was not homogenous, with between one (566 scaffolds) and 34 (1 scaffold) markers per scaffold reflecting variation in scaffold length and marker density across the genome. Overall, 349 scaffolds contained 2 unique markers whereas 193 and 142 scaffolds contained three and four unique markers respectively (Fig. 4b). A positive correlation between scaffold length and number of markers was detected (Spearman’s Rank correlation ρ = 0.68, P = 0.01).

#### Development of a consensus physical map of pseudo-chromosomes

Seven macadamia maps were employed in ALLMAPS to generate a consensus scaffold order (physical map) attributed to individual chromosomes by anchoring the marker sequences to the *M. integrifolia* ‘741’ v2 genome scaffolds. The consensus map included 4,184 markers (Table 1) with a mean marker density of 8.1 markers per Mb. Under the optimal weight regime, 69.7% (519.2 Mb) of the assembly was anchored to 14 linkage groups. In total, 1,465 scaffolds were anchored of which 474 were N50 scaffolds. Of the anchored scaffolds 609 had a single marker, while 856 had ≥2 markers and could be used for orientation. All scaffolds greater >1 MB (57) were anchored, as were 94% of the scaffolds with sequence lengths between 50 kb and 1 MB. Of the unanchored scaffolds 820 were less than 10,000 bp, of which 682 were less than 5,000 bp.

The ‘741’ self map alone anchored 33.3% of the genome assembly. Although constructed entirely with heterozygous SNP markers from the single cultivar, this map anchored 550 scaffolds, of which 246 were N50 scaffolds. Of these, 176 scaffolds included ≥2 markers (Table 1, Fig. 4c).

Successively combining multiple male and female maps from the biparental populations led to a progressive increase in the proportion of the genome assembly that was anchored. The ‘741’ paternal map anchored 29% of the genome, including 213 N50 scaffolds and 121 scaffolds anchored with ≥2 markers. Maternal maps from the ‘741’ x ‘A4’ and ‘741’ x ‘A268’ crosses anchored 39.9% and 32.8% of the assembly with 687 and 543 scaffolds respectively.

Compared with the *M. integrifolia* ‘741’ maps, parental maps derived from the *Macadamia* interspecific hybrid cultivars ‘A4’ and ‘A268’ included relatively more markers and anchored and oriented a greater proportion of the genome assembly (Fig. 4c). The ‘A4’ paternal map anchored more of the genome assembly than any other map alone (50.4%), with 381 scaffolds having ≥2 markers. The paternal ‘A268’ map anchored 336 scaffolds with ≥2 markers on (Table 1, Fig. 4c).

Congruence between the physical consensus map and the genetic linkage maps was estimated in ALLMAPS. Overall, there was high congruence between the individual and consensus maps with average congruence (Spearman’s Rank correlation coefficient ρ > 0.78, p < 0.01) for each of 14 linkage groups and (ρ > 0.90,p < 0.01) for ten linkage groups (Table 2).

**Table 2:** Spearman’s Rank correlation coefficient of marker congruence between individual linkage maps and the ALLMAPs consensus map, and average correlation for each linkage group and map. *P < 0.05, **P < 0.01.

LG	Mb	Map 1 (741×741)	Map 2 741 ♀ (741xA4)	Map 3 741♀ (741xA268)	Map 4 741♂ (A268x741)	Map 5 A4♂ (741xA4)	Map 6 A268♂ (741xA268)	Map 7 A268♀ (A268x 741)	LG Average
		n = 116	n = 51	n = 35	n = 97	n = 51	n = 35	n = 97	
LG1	29	0.99**	0.94**	0.90**	0.85**	0.90**	0.94**	0.89**	**0.92**
LG2	47	1.00**	0.94**	0.95**	0.20	0.94**	0.98**	0.91**	**0.85**
LG3	37	0.98**	0.97**	0.91**	0.96**	0.98**	0.98**	0.60**	**0.91**
LG4	35	0.95**	0.96**	0.94**	0.98**	0.92**	0.80**	0.85**	**0.91**
LG5	44	0.99**	0.94**	0.54**	0.93**	0.98**	0.95**	0.81**	**0.88**
LG6	38	0.97**	0.90**	0.93**	0.94**	0.96**	0.87**	0.90**	**0.92**
LG7	38	1.00**	0.97**	0.99**	0.96**	0.94**	0.95**	0.66**	**0.92**
LG8	42	0.98**	0.90**	0.95**	0.92**	0.94**	0.95**	0.95**	**0.94**
LG9	36	0.99**	0.96**	0.95**	0.95**	0.73**	0.92**	0.85**	**0.91**
LG10	41	0.97**	0.96**	0.84**	0.90**	0.90**	0.94**	0.94**	**0.92**
LG11	32	0.99**	0.88**		0.76**	0.98**	0.91**	0.91**	**0.90**
LG12	33	1.00**	0.82**	0.78*	0.81**	0.86**	0.67**	0.50**	**0.78**
LG13	34	0.98**	0.96**	0.90**	0.98**	0.91**	0.82**	0.88**	**0.92**
LG14	34	1.00**	0.99**	0.95**	0.95**	0.73**	0.92**	0.86**	**0.91**
Map Average		**0.98**	**0.93**	**0.89**	**0.86**	**0.90**	**0.90**	**0.82**	**0.90**

The weakest average congruence was observed for LG12 (ρ = 0.78), and appeared due largely to incongruence of the hybrid ‘A268’ maps (Map 6, ρ = 0.67 and Map 7, ρ = 0.50) and with Map 3 (ρ = 0.78). The lowest congruence with the consensus map was LG02 of paternal ‘741’ map (ρ = 0.20). The maternal ‘A268’ map had the weakest mean correlation overall with the consensus map (ρ = 0.82) primarily due to low correlations at LG03, LG07 and LG12 (Table 2, Table S3).

ALLMAPS was also used to anchor the ‘741’ v2 assembly by using both of the integrated cultivar maps from ‘741’ and ‘A268’. The integrated ‘741’ map anchored 42.8% (754 scaffolds, 279 with ≥2 markers) and the integrated ‘A268’ map anchored 51.7% (920 scaffolds, 389 with ≥2 markers) (Table 1, Fig. 4c).

**Figure 5:**
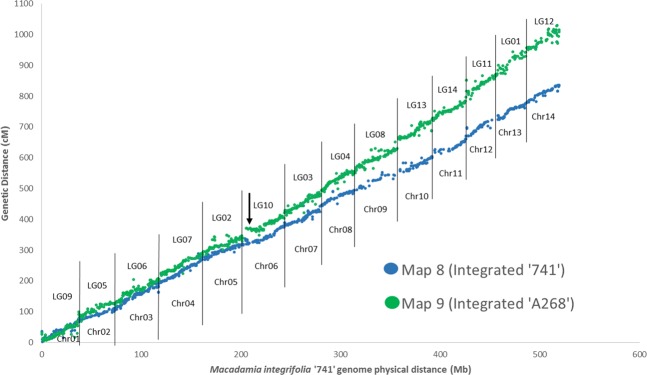
Marker positions in the integrated ‘741’ and ‘A268’ linkage maps relative to the *M. integrifolia* ‘741’ genome. The X axis is physical distance in chromosome order. The Y axis is genetic distance in centimorgans (cM). Vertical lines indicate the boundaries between linkage groups (LG). and chromosomes (Chr). Arrow indicates location of possible inversion.

In order to compare physical and genetic linkage distances the integrated ‘741’ and ‘A268’ maps were plotted on the reconstructed chromosomes of *M. integrifolia* cultivar ‘741’ (Fig. 5). Although the integrated ‘A268’ map anchored more of the genome due to higher marker density than the integrated ‘741’ map, several linkage groups showed weak congruence between the integrated ‘741’ and integrated ‘A268’ maps (Fig. 5).

There was relatively strong congruence between ‘741’ and ‘A268’ maps for LG09, 05, 06, 07, and 02, with alignments in ALLMAP plots contributing to a single, or close to single, diagonal line (Fig. 5). The remaining linkage groups showed regions of divergence from the main diagonal with the most markers diverging from a linear alignment in the ‘A268’ map. The greatest departures from collinearity were observed within LG10, LG11, LG01 and LG12, with outlying markers located at a greater distances from the main diagonal. Linkage groups 11, 01 and 12 were placed at the end of the reconstructed genome assembly as Chromosomes (Chr) 12, 13 and 14 respectively. A section of LG10 (Chr06) on the ‘A268’ map was perpendicular to the main diagonal of the ‘741’ genome, which may indicate a possible inversion (Fig. 5). It is important to note that the genome assembly was constructed from ‘741’ sequence data.

## Discussion

The aim of this study was to establish a set of reference genetic linkage maps for macadamia with marker density suitable for constructing a chromosome-scale assembly. For this primarily outcrossing perennial tree crop, we made use of four segregating populations to construct a series of linkage maps that made maximised use of pseudo-testcross segregation patterns available within full-sib, biparental and self-polinated progeny. The outcome of this combined linkage analysis facilitated the generation of a consensus physical map. All linkage maps generated had 14 linkage groups, corresponding to the haploid chromosome number of *Macadamia*^3^. The production of integrated cultivar maps from multiple parental linkage maps increased marker density compared with component maps. Overall, the combination of linkage maps and pseudo-chromosome assemblies presented here represent a substantial advance in macadamia genetics and genomics.

### Advantages of multiple populations for genetic linkage mapping

In order to generate accurate information to meet the requirements for marker-assisted trait breeding and genome anchoring, contemporary linkage maps require high marker density to increase map resolution and statistical support for marker order^35^. Traditionally, linkage mapping has taken advantage of tracking segregation of alleles from parents that diverge for a certain phenotype or trait. However, limiting this exercise to just two parents often limits the genetic (allelic) variation and informative markers available, making it difficult to produce high density genetic linkage maps^36^. While larger population sizes are expected to increase the number of detectable recombination events, the use of multiple populations with differing parentage can increase the numbers of markers detected and map density^13^.

The value of including multiple populations into mapping projects to capture allelic diversity and increase representation of recombination has been recognised and demonstrated both for inbreeding species such as *Arabidopsis*^37^ and rice^38,39^, as well as outcrossing crops including apple^40,41^, strawberry^42^ and peach^13^. This includes Multi-parent Advanced Generation Intercross (MAGIC) populations which have been developed for a number of inbreeding crops such as tomato^43^, wheat^44^ and sorghum^45^ in order to increase genetic diversity and sample additional recombination events and subsequently improve map resolution.

The relatively small population sizes used in this study (n = 35 to 116) limited the number of COs detected for each population. However, the inclusion of three biparental populations with different sets of informative markers increased the overall allelic diversity in the dataset thereby increasing marker density and coverage of the genome.

Strong congruence between component maps has led to successful map integration in other studies^46–48^. Combining information from all six parental maps and the ‘741’ self map generated sufficiently high level of congruence (ρ = 0.90, p < 0.01) to allow the generation of integrated maps. The high frequency (>80%) of common markers between parental maps allowed map integration increasing marker density by 63% and 78% in the ‘741’ and ‘A268’ integrated maps compared to individual maps. This is consistent with previous studies that have shown that integrated maps generated from multiple populations can provide higher marker density and therefore greater genome coverage compared to individual maps^13,49^.

### Crossover frequency and recombination varied between maps

Discrepancies between linkage maps, including length and crossover frequency, may reflect variation in recombination rates^50,51^, which has been detected along chromosomes in many plant species^52,53^. Variation in the distribution of CO along chromosomes has been found to arise due to a range of factors, including the distribution of transposable elements, DNA methylation and histone marks, heterozygosity, domestication, environment, age, sex and pathogen infection^54–58^.

Macadamia is preferentially outcrossing but there have been reports of significant differences in the rates of self-fertilisation between cultivars^7,59–61^. Cultivar ‘741’ is relatively self-fertile and paternity testing of the progeny used in this study identified self-fertilised progeny (3.3–5.6%) from controlled crosses where ‘741’ was the female parent but not where ‘A268’ was the female parent (Table S1). Maternal maps generated from ‘741’ had a higher proportion of progeny in each linkage group where no CO’s were detected, compared to the maternal ‘A268’ map, while the converse is observed in the males (Fig. 3). This suggests that differences in the mating systems of the parental cultivars used in this study may have contributed to the observed variation in recombination rates. Mating system (selfing or outcrossing) has been linked to variation in recombination rates, with selfing resulting in a lower degree of effective recombination^62^, and transposable element polymorphism being higher in selfing populations, as in *A. lyrata*^63^. Differences in recombination rate between self and outcrossing species have been reported in some crop species, including tomato^64^ and mushroom^65,66^.

The distribution of gametic COs is also known to be affected by variation in heterozygosity, with localised recombination frequency being dependent on factors including chromatin structure and repair mechanisms^54^. Recombination suppression may also be observed as a result of a heterozygous state in *cis*, with varying homolog polymorphism shaping meiotic recombination^67–69^. Heterozygosity is reportedly high in macadamia cultivars and wild accessions^22,70–74^. In this study, heterozygosities of 0.18 (‘741’, ‘A268’) and 0.16 (‘A4’) were observed in the parental genotypes. Although this was comparable to previous reports also based on codominant DArT data for macadamia breeding populations^19,30^, it is significantly lower than SNP-based estimates for other fruit crops including olive (0.44), apple (0.36) and peach (0.28)^75–77^. For macadamia, the relatively low level of heterozygosity detected in this study may reflect the bottleneck in recent domestication^78^. Based on data from randomly amplified RAMiFi markers, heterozygosity was reported as being lower in the Hawaiian compared to hybrid macadamia cultivars^27^. Interestingly, our results based on 5,793 (‘741’), 4,727 (‘A4’) and 4,867 (‘A268’) SNP markers, excluding those with missing data, indicated little difference in heterozygosity between the Hawaiian ‘741’ and Australian ‘A268’ and ‘A4’ hybrid cultivars.

The mapping populations used in this study included three different parental cultivars (‘741’, ‘A268’ and ‘A4’), with populations established from different crosses of these cultivars. The maps generated provide a range of different map lengths and CO frequencies, depending on the cross. In general, the maps generated from the *M. integrifolia* ‘741’ genotype were relatively consistent. For instance, the proportion of distorted markers and CO frequencies were consistent in the three parental ‘741’ maps. Likewise, the ‘741’ maps are consistently shorter than the parental maps from the hybrid species ‘A268’ and ‘A4’ irrespective of whether the map was derived from maternal or paternal segregation.

In contrast, greater variation in map length, CO frequency and marker distortion was detected in parental maps from the hybrid cultivars. Map length was relatively longer for maternal (3,662.05 cM, 1,701 markers) and paternal (1,261.7 cM, 1,797 markers) ‘A268’ maps. Similarly, the male ‘A4’ map was longer than the corresponding ‘741’ map derived from the same cross. This may suggest that differences in the genetic background may be contributing to the variations between the maps. In grape, variations in recombination rate were detected based on crosses using a range of parents, interspecific crosses and a F1 population generated from two half-sib parents^79^. Likewise, differences in recombination rate attributed to different *Eucalytptus* parents has been found to be statistically significant^80^.

Both of the parental maps from the ‘A268’ x ‘741’ cross had a relatively high frequency of at least two apparent COs per linkage group compared with the other crosses. We also observed that maps generated from crosses involving hybrid genotypes (’A268’ and’A4’) had a higher level of marker clustering, with fewer unique marker positions (Table 1). This may indicate the presence of co-segregating markers and lead to inaccuracies in CO counts^81^.

Cultivar ‘741’ is *M. integrifolia* while ‘A268’ and ‘A4’ are hybrids of *M. integrifolia* and *M. tetraphylla*. These species have a reported overlapping natural distribution of approximately 80 km in the subtropical rainforests of eastern Australia^22^ and readily hybridise both in natural and domesticated settings, indicating that there are no genetic barriers to hybridization. While it is possible that the hybrid nature of ‘A268’ and ‘A4’ may contribute to the relatively greater map length and COs observed, further studies are needed to formulate a more conclusive understanding of the contribution of parental genotype differences to recombination of heritable traits in macadamia.

Variations in map length and CO frequency between maps may also result from genotyping error and this may contribute to the relatively higher level of observed double recombinants detected in the ‘A268’ maternal map. Extensive measures were taken to exclude erroneous markers, however, Mendelian inconsistencies were detected and are indicative of genotyping error in the pre-filtered dataset (Table S2). There is evidence that reduced-representation genome sequencing approaches such as DArT based on restriction digestion can underestimate genetic diversity due to allele dropout (where one or more alleles are not typed) and null alleles^82–85^. Allele dropout is predicted to increase in heterozygous taxa because it is associated with polymorphism at restriction sites^86^.

### Distinct regions of segregation distortion detected on the ‘741’ self map

Apparent SD may emerge due to experimental factors such as sampling error, missing data, and genotyping errors. Conversely it may reflect actual biological factors such as gamete competition, hybrid incompatibility, deleterious alleles (genetic load), selection and even chromosome loss or rearrangements^87–89^. Given sufficient marker density, biologically-derived SD is more likely to be detected as clusters or gradients of markers having similar behaviour within a chromosomal region.

Markers showing distortion have been recorded in a number of crops^90,91^. Markers displaying SD can be useful in QTL mapping^92^ as they are often linked to candidate genes for specific traits. For instance, it was found that regions of SD in sweet cherry, where 8% of markers showed distortion, are linked with hotspots containing fruit and bloom time traits, as well as a self-incompatibility modifier locus^93^. In cotton, several important agronomic traits have been recorded on chromosome 18 where 34% of markers showed distortion^90^.

Within the ’741’ self map we found that many of the markers displaying SD clustered into seven distinct regions (Fig. 2). The proportion of markers with SD (16%), was consistent with the previous genetic linkage maps for macadamia (16 and 20%)^18,27,94^. Peace, *et al*.^18^ identified SD in four regions compared to seven across six linkage groups in the current study. They also reported a higher proportion of distorted markers in the hybrid cultivar ‘A16’ compared to *M. integrifolia* cultivar ‘246’. Likewise, in our study a higher proportion of distorted markers were detected when ‘A268’ was the female parent. The relatively higher levels of distorted markers in the ‘741’ self and ‘A268’ maternal maps may indicate selection against deleterious loci. For regions within some pseudo-chromosomes it appears that there may be evidence for gamete-specific skews in SD.

### Genome anchoring

We have generated the first sequence-based linkage maps for macadamia. Compared with the individual maps, the combination of genetic maps generated from different mapping populations improved the proportion of the genome sequence assembly anchored (Table 1). The high congruence between maps and their effectiveness in anchoring genome scaffolds are strong indicators of map reliability. Accurate anchoring of genome scaffold sequences to generate pseudo-chromosome assemblies requires high quality of both scaffold sequences and genetic linkage maps. All markers (4,266) that mapped to at least one of the nine genetic linkage maps were aligned by BLASTn to the macadamia v2 genome scaffolds. Of these, 83% mapped to a single scaffold and 12% to two locations, while the remainder (5%) mapped to multiple scaffolds (Fig. 4a). These results are consistent with a recent study using the same draft genome where 80% of SNPs mapped to a single scaffold^19^.

The ALLMAPS scaffold ordering method maximises collinearity to generate assemblies that are consistent with multiple maps^34^ and has been used for anchoring genome assemblies using linkage maps for a range of crops including capsicum, cucumber, foxtail millet and cocao^95–97^. The genetic linkage maps developed in this study anchored 1,465 scaffolds covering 69.7% of the macadamia genome assembly. This facilitated the construction of 14 chromosome-scale sequence assemblies with a total length of 519.3 Mbp and anchoring 85% of N50 scaffolds. Scaffold orientation requires at least two markers per scaffold. Of the 1,465 scaffolds anchored in the consensus physical map 58% contained ≥2 markers, including all scaffolds over 1 Mbp in length.

As expected, congruence of marker order was generally high between each of the self and parental maps and the consensus physical map. However, a relatively lower congruence (0.89, 0.82) between the consensus and maps generated from the ‘A268’ x ‘741’ population may indicate local structural variations in the ‘A268’ genome (Table 2, Fig. 5). It is possible that structural variation among the genomes of the parents of mapping populations used in this study has contributed to variation in the recombination rates between progeny of different populations such as has occurred in *Arabidopsis*^98^, banana^99^ and cotton^100^.

### Potential genome-specific chromosomal rearrangements

Using sequence-based markers to compare the genetic maps of different genotypes against a physical genome sequence can provide information on potential chromosomal rearrangements and smaller structural variations^40,101,102^. The observed variation in recombination data between the integrated ‘741’ and ‘A268’ maps relative to the ‘741’ genome assembly are indicative of structural rearrangements in ‘A268’. Of particular note is a region at the top of Chr06 (LG10) where a section of the ‘A268’ map forms a close to perpendicular line from the genome diagonal indicating a possible terminal inversion on Chr06 (Fig. 5).

SSR-based estimates of genetic distance within the macadamia species-complex indicate historical separation between the *M. integrifolia* and *M. tetraphylla* gene pools^26,72^. However, there are currently no estimates of the likely timing of this divergence. Peace^27^ conducted the most extensive genetic analysis to date that included representatives of all four *Macadamia* species and industry cultivars, using randomly-amplified markers. Of all macadamia cultivars grown in Australia the highest proportion of *M. tetraphylla* was reported in ‘A268‘, consistent with an F1 interspecific hybrid. The genotype of the other hybrid cultivar ‘A4’ included in this study is consistent with its reported parentage and indicates that ‘A4’ contained approximately 25% *M. tetraphylla*^27^. Hybridisation and differences in the respective genome composition of the parental cultivars used in this study may underly observed variations in genome structure and recombination rate between ‘741’, *M. integrifolia* (100%) and the hybrids: ‘A268’, *M integrifolia* (50%) x *tetraphylla* (50%), and ‘A4’, with parents ‘Own Choice’, *M. integrifolia* (100%) x ‘Renown’, *M. integrifolia* (50%) x *M. tetraphylla* (50%).

## Conclusions

The strategy of amalgamating information from multiple populations and linkage maps was successful in increasing marker density and detecting variation in CO frequency between parental genotypes. Compared with a single-species biparental population, the use of four populations involving a common parent and two additional parents of diverse origin both increased the number of recombination events and informative markers. Generating integrated cultivar-specific maps also enabled us to resolve inconsistencies in marker order and reduce gaps in individual maps. Combining genetic maps to facilitate generation of a consensus physical map was successful and improved the proportion of the assembly anchored in comparison to individual maps.

In conclusion, we demonstrate the value of establishing a series of genetic linkage maps and reconstructed genome as a platform for future genetic and genomic studies in the perennial outcrossing nut tree macadamia. The genetic linkage maps generated in this study were successfully utilised for anchoring and orientation of genome scaffolds and construction of the first pseudochromosome-scale assembly for macadamia. This represents a significant increase in our understanding of the genetic landscape and genome for this nut crop. The set of maps, large number of sequence-based markers and the reconstructed genome provide a toolkit to underpin future breeding that should help to extend the macadamia industry as well as provide resources for the long term conservation of natural populations in eastern Australia of this unique Proteaceous genus.

## Supplementary information

Supplementary Information.

## Data Availability

Supplementary Data (Table S1 to S5) are included as additional online resources to the manuscript. The v2 assembly scaffolds used for genome anchoring are available in the EMBL-ENA repository [assembly accession ERS2953073]. Computational data from “Maximising recombination across macadamia populations to generate linkage maps for genome anchoring” including genetic linkage map data, charts and the ALLMAPS golden path file detailing scaffold order on pseudo-chromosomes have been deposited in the Southern Cross University data repository [http://dx.doi.org/10.25918/5dc2589924ca2].
