# Supplementary material for: Maximising recombination across macadamia populations to generate linkage maps for genome anchoring
Source: Sci Rep. 2020 Mar 19;10:5048. doi: 10.1038/s41598-020-61708-6 (PMC7081209; doi:10.1038/s41598-020-61708-6)
Supplement: Supplementary file 1 — Supplementary Information. [file 41598_2020_61708_MOESM1_ESM.pdf]

Supplementary Information

Maximising recombination across macadamia populations to generate linkage maps for genome anchoring.

Kirsty S Langdon, Graham J King, Abdul Baten, Ramil Mauleon, Peter C Bundock, Bruce L Topp and Catherine J Nock

Table S1. Paternity assignment of progeny from mapping populations using SNP markers

Putative Cross	n	Cross	# based on paternity assignment	% expected cross	% Self	% unexpected cross
**741 x A268**	60	741xA268	34	56.7	3.3	43.3
		741xA4	23			
		741x741	2			
		unassigned	1			
**741 x A4**	36	741xA4	34	94.4	5.6	5.6
		741x741	2			
**A268 x 741**	97	A268x741	90	92.8	0	7.2
		unassigned	7			
**741 x 741**	70	741x741	49	70	70	30
		unassigned	21			

n number of progeny

Table S2. Data filtering parameters and statistics for codominant SNP and dominant PAV markers

**Markers**	**Codominant SNP**								**Dominant PAV**		**SNP and PAV**										
Section	1. Segregating SNP		2. parental genotype inconsistency			3. Mendelian inconsistency			4. Segregating PAV		5. SNP and PAV	6. Missing data >10%			7. PIC, call rate, reproducibility			8. Segregation type			9. Mapped Markers
Cross	proportion of total SNPs	number	proportion of available SNPs	excluded	retained	proportion of available SNPs	excluded	retained	proportion of total PAVs	number	available	proportion of available markers	excluded	retained	proportion of available markers	excluded	retained	1:2:1	1:1	3:1	number of markers mapped
741 x 741	0.18	1069	0.06	64	1005			1005		0	1005	0.03	30	975	0.075	74	901	901	0	0	884
741 x A4	0.40	2440	0.12	293	2147	0.47	1009	1138	0.37	3703	4841	0.12	581	4260	0.14	587	3673	132	654	2887	3139
741 x A268	0.38	2318	0.15	348	1970	0.47	926	1044	0.36	3585	4629	0.14	648	3981	0.11	448	3533	131	590	2812	2674
A268 x 741	0.38	2318	0.15	348	1970	0.47	926	1044	0.36	3585	4629	0.25	1157	3472	0.24	822	2650	128	404	2118	2395

**Section**	**Marker filtering**
1	Identification of informative segregating codominant SNP markers
2	Markers filtered based on inconsistency between parental replicates
3	Markers filtered based on Mendelian inconsistency in progeny given parental genotypes
4	Identification of informative segregating dominant PAV markers
5	Available and informative SNP and PAV markers
6	Markers filtered with >10% missing data
7	Markers filtered with <90% reproducibility, <90% call rate, <0.25 polymorphic information content (PIC)
8	Segregation type of available markers
9	Final number of markers mapped

Table S3. Heat map of coupling and repulsion phase marker ratios

LG	Map 2	Map 3	Map 4	Map 5	Map 6	Map 7
1	1.5	3.5	10.0	1.9	3.4	5.6
2	1.9	2.6	1.6	1.5	2.9	8.6
3	1.8	1.9	1.5	1.8	2.5	1.9
4	1.7	2.0	1.4	1.9	5.1	2.7
5	1.8	1.1	2.1	1.4	2.2	2.9
6	1.3	1.5	1.4	1.8	3.5	2.0
7	1.3	0.8	2.7	1.5	3.3	2.2
8	1.5	1.4	2.6	1.7	2.7	3.3
9	1.7	2.0	2.5	2.3	3.3	6.4
10	1.7	1.8	2.7	2.6	3.2	2.9
11	1.3	1.5	1.4	2.1	0.7	2.2
12	3.3	N/A	5.5	2.1	7.7	12.3
13	1.0	2.5	1.3	1.9	2.8	2.1
14	0.7	2.3	N/A	2.0	5.3	4.5
**Average**	**1.6**	**1.9**	**2.8**	**1.9**	**3.5**	**4.2**

**Map**	**Cross**
2. 741 ♀	741 x A4
3. 741♀	741 x A268
4. 741♂	A268 x 741
5. A4♂	741 x A4
6. A268♂	741 x A268
7. A268♀	A268 x 741

Table S4. Spearman's rank correlation co-efficient of marker order between genetic linkage maps

	Map 1 v Map 2		Map 1 v Map3		Map 1 v Map4		Map 1 v Map 8		Map 5 v Map 6		Map 5 v Map 7		Map 6 v Map 7	
LG	ρ	n	ρ	n	ρ	n	ρ	n	ρ	n	ρ	n	ρ	n
1	0.83**	20	0.94**	9	0.98**	9	1.00**	70	0.98*	8	0.95**	13	0.88**	36
2	0.97**	12	0.97**	11	0.97**	9	1.00**	67	0.96**	69	0.84**	52	0.86**	121
3	0.94**	16	0.97**	18	0.97**	14	1.00**	54	0.98**	42	0.60**	28	0.65**	131
4	0.98**	23	0.94**	20	0.98**	16	0.99**	73	0.82**	61	0.80**	61	0.68**	152
5	0.99**	20	0.22	16	0.77*	12	1.00**	62	0.94**	16	0.87*	10	0.87**	123
6	0.93**	13	0.62	10	0.99**	10	1.00**	89	0.83**	59	0.91**	40	0.85**	116
7	0.99**	28	0.97**	19	0.80**	14	1.00**	73	0.86**	20	0.67*	12	0.71**	98
8	0.91**	21	0.98**	13	0.69	9	0.98**	23	0.98**	45	0.92**	32	0.94**	136
9	0.99**	20	0.74*	12	0.97*	8	1.00**	63	0.52**	27	0.03	16	0.95**	118
10	0.85**	13	0.49	13	0.44	12	1.00**	62	0.86**	64	0.91**	34	0.92**	101
11	0.97**	11	1.00**	11	0.20	4	1.00**	56	0.96	7	0.92**	39	0.93**	11
12	0.87**	12	0.71*	11	0.00	5	1.00**	54	N/A	2	0.30	14	0.23	21
13	0.96**	11	0.93	7	1.00	5	0.97**	37	0.91**	47	0.79**	37	0.80**	98
14	0.85*	9	1.00	6	1.00	5	0.99**	39	0.93**	47	0.92**	26	0.96**	128
**Average**	**0.93**		**0.82**		**0.77**		**1.00**		**0.82**		**0.75**		**0.80**	
**Total**		**229**		**176**		**132**		**822**		**514**		**414**		**1390**

**Map**		**Cross**		
			ρ	Spearman's Rank correlation
Map 1	741 Self	741 x 741	n	Number of common markers
Map 2	741 ♀	741 x A4	*	P < 0.05
Map 3	741♀	741 x A268	**	P < 0.01
Map 4	741♂	A268 x 741	1.00	Perfect Correlation
Map 5	A4♂	741 x A4	0.9 < 1.0	Very High Correlation
Map 6	A268♂	741 x A268	0.7 < 0.9	High Correlation
Map 7	A268♀	A268 x 741	0.5 < 0.7	Moderate Correlation
Map 8	Integrated 741		0.3 < 0.5	Low Correlation

Table S5. Summary of apparent chiasmata across linkage groups for each map.

**Cross**	**741 x 741**					**741 x A4**										**741 x A268**										**A268 x 741**									
**Progeny**	**116**					**51**										**35**										**97**									
	**Map 1. '741' Self**					**Map 2. 741 ♀**						**Map 5. A4♂**				**Map 3. 741♀**					**Map 6. A268♂**					**Map 7. A268♀**					**Map 4. 741♂**				
LG	0	1	2	3	≥ 4	0	1	2	3	≥ 4	0	1	2	3	≥ 4	0	1	2	3	≥ 4	0	1	2	3	≥ 4	0	1	2	3	≥ 4	0	1	2	3	≥ 4
1	34	50	31	1	0	23	19	7	1	1	22	15	2	5	7	20	9	2	4	0	25	10	0	0	0	38	21	19	11	8	35	56	6	0	0
2	16	59	32	8	1	24	20	5	1	1	15	19	9	3	5	21	12	1	1	0	15	16	3	1	0	29	13	23	6	26	29	17	16	17	18
3	22	61	30	3	0	23	20	1	6	1	11	27	5	5	3	12	15	1	6	1	14	14	2	5	0	29	6	16	16	30	30	37	14	6	10
4	31	46	32	7	0	24	15	5	3	4	20	16	6	5	4	18	11	2	4	0	16	8	8	1	2	26	16	13	11	31	38	30	12	6	11
5	32	50	30	3	1	23	23	1	3	1	16	18	8	4	5	18	4	8	4	1	18	11	3	2	1	26	20	24	12	15	38	16	17	13	13
6	20	44	41	11	0	18	21	3	6	3	12	21	8	7	3	14	15	2	4	0	14	5	8	2	6	26	20	15	15	21	36	29	12	9	11
7	26	61	27	2	0	20	26	3	0	2	18	23	8	1	1	21	13	0	1	0	17	10	1	6	1	29	18	19	12	19	37	24	18	11	7
8	18	60	32	6	0	28	11	6	1	5	12	21	11	4	3	18	12	2	3	0	16	8	5	4	2	19	27	8	24	19	33	33	18	7	6
9	17	55	32	11	1	19	25	2	4	1	18	8	14	4	7	13	16	2	4	0	10	19	2	3	1	28	20	17	25	7	26	36	24	7	4
10	33	41	34	8	0	22	20	4	4	1	21	16	11	1	2	16	5	13	1	0	19	10	2	2	2	32	24	11	14	16	28	31	23	9	6
11	27	63	23	3	0	26	18	3	3	1	20	18	5	5	3	16	19	0	0	0	21	13	0	1	0	21	27	22	12	15	38	38	15	5	1
12	24	53	35	4	0	23	10	13	3	2	20	13	10	6	2	30	3	1	1	0	33	2	0	0	0	27	12	21	10	27	29	27	27	7	7
13	36	49	25	6	0	32	19	0	0	0	20	12	15	3	1	16	7	8	3	1	17	8	7	2	1	30	31	10	18	8	41	36	12	5	3
14	32	66	18	0	0	20	25	5	1	0	16	11	13	6	5	19	15	1	0	0	11	21	2	1	0	38	23	13	8	15	43	48	4	2	0
**Total**	**368**	**758**	**422**	**73**	**3**	**325**	**272**	**58**	**36**	**23**	**241**	**238**	**125**	**59**	**51**	**252**	**156**	**43**	**36**	**3**	**246**	**155**	**43**	**30**	**16**	**398**	**278**	**231**	**194**	**257**	**481**	**458**	**218**	**104**	**97**
**Proportion**	**0.2**	**0.5**	**0.3**	**0.0**	**0.0**	**0.5**	**0.4**	**0.1**	**0.1**	**0.0**	**0.3**	**0.3**	**0.2**	**0.1**	**0.1**	**0.5**	**0.3**	**0.1**	**0.1**	**0.0**	**0.5**	**0.3**	**0.1**	**0.1**	**0.0**	**0.3**	**0.2**	**0.2**	**0.1**	**0.2**	**0.4**	**0.3**	**0.2**	**0.1**	**0.1**

LG Linkage group
